# A specialized face-processing model inspired by the organization of monkey face patches explains several face-specific phenomena observed in humans

**DOI:** 10.1038/srep25025

**Published:** 2016-04-26

**Authors:** Amirhossein Farzmahdi, Karim Rajaei, Masoud Ghodrati, Reza Ebrahimpour, Seyed-Mahdi Khaligh-Razavi

**Affiliations:** 1 School of Cognitive Sciences (SCS), Institute for Research in Fundamental Sciences (IPM), Tehran, Iran; 2 Department of Physiology, Monash University, Melbourne, VIC, Australia; 3 Department of Computer Engineering, Shahid Rajaee Teacher Training University, Tehran, Iran; 4 Computer Science and Artificial Intelligence Laboratory, Massachusetts Institute of Technology, USA

## Abstract

Converging reports indicate that face images are processed through specialized neural networks in the brain –i.e. face patches in monkeys and the fusiform face area (FFA) in humans. These studies were designed to find out how faces are processed in visual system compared to other objects. Yet, the underlying mechanism of face processing is not completely revealed. Here, we show that a hierarchical computational model, inspired by electrophysiological evidence on face processing in primates, is able to generate representational properties similar to those observed in monkey face patches (posterior, middle and anterior patches). Since the most important goal of sensory neuroscience is linking the neural responses with behavioral outputs, we test whether the proposed model, which is designed to account for neural responses in monkey face patches, is also able to predict well-documented behavioral face phenomena observed in humans. We show that the proposed model satisfies several cognitive face effects such as: composite face effect and the idea of canonical face views. Our model provides insights about the underlying computations that transfer visual information from posterior to anterior face patches.

Face recognition is robustly performed by human and non-human primates despite many transformations in size, position, and viewpoint of faces. The mechanism of face processing has been extensively studied in different species using different recording techniques^1^,^2^,^3^,^4^,^5^,^6^,^7^,^8^,^9^. Selective neuronal activities to faces have also been reported in other non-human primate such as chimpanzees^10^,^11^ and other species including dogs^12^,^13^ and sheep^14^,^15^. This indicates the crucial role of face processing in understating the ongoing cognitive processes in the brain. Electrophysiological and functional imaging studies have shown that faces are processed through specialized networks in human and non-human primate’s brain^3^,^4^,^5^,^6^, meaning that a particular mechanism is involved in face processing. In addition, there are several face-specific perceptual phenomena such as Composite Face Effect (CFE)^16^,^17^,^18^, Inversion Effect (IE)^18^,^19^,^20^,^21^, and Other-Race Effect (ORE)^22^,^23^,^24^, only applicable to face images.

Functional Magnetic Resonance Imaging (fMRI) in monkeys has revealed six discrete face-selective regions, consisting of one posterior face patch [posterior lateral (PL)], two middle face patches [middle lateral (ML) and middle fundus (MF)], and three anterior face patches [anterior fundus (AF), anterior lateral (AL), and anterior medial (AM)], spanning the entire extent of the temporal lobe^3^. Each region has a different role in face processing. Cell recording from neurons in these areas of monkey brain suggests a functionally hierarchical organization for face processing^4^. First in the hierarchy is PL, which contains many face-selective cells, driven by the presence of face components^25^. Middle patches represent simple properties of faces (e.g., face-views) and in anterior parts, neurons become selective to more complex face properties (e.g., face identities^4^).

Many behavioral studies have also indicated that face images are specific for the visual system compared to other objects^8^,^26^. Face specificity may arise from learning during social interactions. We are all expert face-processing agents, and able to identify very subtle differences within the category of faces, despite substantial visual and featural similarities. Identification is performed rapidly and accurately after viewing a whole face. This refers to a hotly-debated, yet highly-supported concept, known as holistic face processing. The composite face effect (CFE) illustrates that face perception is performed through integration of different face parts as a whole^17^. The composite effect is used as an evidence to support holistic face processing. Another support for holistic processing is the inversion effect (IE). Subjects’ performances in face identification drop significantly when inverted faces are presented, which is a result of configuration loss^27^,^28^.

Humans are better at recognizing faces of own race than other races (other race effect-ORE); this is another well-studied effect in the face literature^23^,^24^. The theoretical underlying computation of this phenomenon is now a matter of debate. One explanation about the other-race effect indicates that it would originate from lack of ability to process configural information of other race faces^23^,^24^. Because we have more experience with faces of our own race, the space of configural face features are better tuned to discriminate faces of own race than faces of another race—to which it has not been extensively exposed. Configural representation seems to be one of the advantages of holistic face processing^23^,^29^. These effects seem to be arisen from the underlying properties of the face processing system that is presumably developed during the learning. On the other hand, there are some effects such as canonical face view that seem to be rooted in stimulus space and are not deemed as inherent properties of face processing system itself. Therefore, effects such as canonical face view may emerge from learning procedures of different models, even if the model is not mimicking the visual hierarchy. The idea of canonical face view refers to the observation that specific face views carry higher information about face identities; therefore, face identification performance for these views is significantly higher^30^,^31^,^32^.

There is a broad support for a general class of computational models based on the hierarchical organization of the visual pathway (reviewed in:^33^,^34^,^35^,^36^). These models have tried to simulate the selectivity and tolerance that exist throughout the visual hierarchy^37^,^38^,^39^. However, several studies have revealed that although such a class of hierarchical models are partially successful, they fail to explain certain properties of human object recognition^40^,^41^; and that deep supervised architectures provide a much better explanation of visual object processing^42^,^43^,^44^,^45^. Recent modeling studies have tried to implement some face-specific properties^46^,^47^. They have been considerably successful in face processing and have been able to explain some face-related phenomena such as, invariance and holistic face processing. However, the underlying computational mechanism of face processing and what happens in face-specific areas (i.e., face patches) has remained unknown. Our proposed model substantially extends previous developments, and reaches an ideal level in which it simulates neural response characteristics of monkey face patches; and explains several behavioral phenomena observed in humans.

The proposed model is based on recent electrophysiological evidence in monkey face selective areas^3^,^4^,^7^. The model has several layers with an organization similar to that of the hierarchical structure of the face processing system. Layers of our model simulate different aspects of face processing and its representational space similar to that of monkey face patches^4^. The model has view selective and identity selective layers consistent with physiological and psychophysical data.

To evaluate the ability of the model in simulating representational space of faces in face-selective areas in the visual system, we compare the model responses with neuronal and some challenging behavioral data. The model is compatible with electrophysiological data for face identification, and the representational geometries of the model layers have characteristics similar to that of ML/MF and AM patches in monkeys.

One of the most important challenges in visual neuroscience is linking the neural responses with behavior. Here, by means of computational modeling, we bridged the gap between these two: we simulated neural responses in a model that reflects/mimics face-specific behavioral effects. Taken together, the results of multiple experiments and comparisons suggest that the proposed model simulates the available cell recording data from monkey face patches and very well explains human behavioral face specific phenomena. This also lends additional support to the idea that man and monkey share several characteristics in processing faces and objects^48^.

## Materials and Methods

### Model Overview

**Figure 1:**
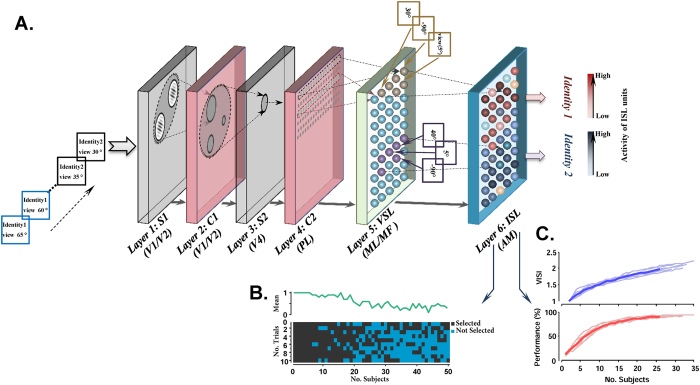
Schematic of the proposed model. (**A**) Each block shows a layer of the model with their properties. S1 and C1 layers represent bars and edges similar to V1/V2 in the visual system. Face parts are represented through S2 and C2 layers. Subsequently, face views are coded in VSL and face identities are coded within the pattern of activities in ISL units (e.g. red circles for Identity 1 and blue circles for identity 2– different shades of red/blue indicate the level of activity). (**B**) Number of selected subjects in ISL during learning: The horizontal axis shows the number of ISL units (No. Subjects) and the vertical axis depicts the number of trials. The green curve shows the average of selected units across 10 random trials. (**C**) VISI and identification performance saturation during learning: The horizontal axis depicts the number of selected ISL units (No. Subjects) and the vertical axis illustrates performance and VISI. The pale curves indicate 10 random runs and the thick (blue and red) curves indicate the average.

The proposed model has a hierarchical structure with 6 processing layers, agreeing well with the hierarchy of the ventral visual pathway and face patches in monkey’s brain (starting from posterior area (PL) to middle parts (ML/MF) and extending to the most anterior part AM). The first four layers of the model extract primary visual features, such as edges and more complex visual patterns. In terms of architecture, these layers are identical to the first four layers of the HMAX model, a biologically plausible model for object recognition^37^. The model was trained with face images from NCKU dataset. The last two layers of the proposed model simulate middle (ML/MF) and anterior (AM) face patches in monkey IT cortex, consistent with electrophysiological data in^3^,^4^. These two layers are called view selective layer (VSL-simulating middle patches, ML/MF) and identity selective layer (ISL-simulating AM). Figure 1A schematically shows the properties of each layer. Figure 1B,C indicate the learning procedure during training and evaluation phases. Figure 1B shows the number of subjects selected during the learning procedure across different trials. As shown in the color-coded pattern, more units are added to the ISL at the beginning of the learning procedure compared to later stages where number of face identities presented to the model is increased. Identification performance and View-invariant Identity Selectivity Index (VISI)–VISI is described in *Materials and Methods* section–were used as the criteria to decide whether new units should be added to the model, Fig. 1C.

### Primary feature extraction layers (S1, C1, S2, and C2)

The first two consecutive layers, S1 and C1, simulate simple and complex cells in the early visual cortex. S1 units are tuned to oriented bars within their receptive field (RF), similar to simple cells in the visual cortex^49^,^50^,^51^. C1 units create slight invariant responses to scale and position of their preferred stimuli using a local max pooling operation over S1 units of the same orientation but different positions and scales^37^.

The subsequent layer is S2. Units in this layer receive their inputs from small portions in C1 responses. The units are selective to the particular prototypes that are randomly extracted from training images in the learning phase. Each prototype is set as the preferred stimulus of a neuron/unit in the S2 layer, the more similar the input image to the prototype, the stronger the responses generated in S2 units. Each prototype is set as the center of a Gaussian-like function in which the distance of input image is calculated relative to the center, equation 1:


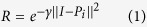


Where *R* is the output response, γ is the sharpness of the tuning function, *I* is the input image and *P* is the extracted prototype. We implemented 1,000 S2 units.

Each unit in the next layer, C2, performs a global max pooling over S2 units with the same prototype in various positions and scales. C2 output is a feature vector for every input image, elements of which explain the degree of similarity between prototypes and the input image. S2 and C2 units have larger receptive fields and are selective to more complex patterns than simple bars and edges. These layers simulate the responses of V4 and anterior IT neurons (PL in monkey cortex).

### View Selective Layer (VSL)

Units in the view selective layer (VSL) receive their inputs from C2 layer through Gaussian tuning functions. Each unit in VSL responds to a specific pattern of C2 responses. For example, for any input image a vector of C2 values (i.e. 1000 C2 features) is compared with a set of vectors that are centers of Gaussian functions in the view selective units. These centers are tuned during the learning phase to different face views (see *Learning procedure*). In this way, different face views are represented over a population of VSL units. Each input image, from evaluation and test dataset, is represented over VSL units, using approximately 300 units (this number may change depending on the learning). The tuning properties in VSL units is inspired by ML/MF neurons in monkey face patches, which are selective to the face view^4^.

### Identity Selective Layer (ISL)

Units in the identity selective layer (ISL) pool inputs through max operation, increasing invariance to the face views. Components in this layer receive connections from several VSL units with different view selectivity. The connections between VSL and ISL units are built up in the learning phase (described in the next section: *Learning procedure*). This is done by correlating face views of the same identity across time (temporal correlation); the idea being that in the real world, face views of an identity smoothly changes in time (abrupt changes of view are not expected). The time interval between face views of two identities (sequence of showing two identities) causes VSL units to make connections with different ISL units. Thus, VSL units with the same identity should be connected to one ISL unit.

View independent identity information is coded in a population of neurons in the AM face patch in monkeys. Consistently, face identities and views of novel subjects create a specific pattern of activities in the ISL units (less than 50 units in our experiments is created during the learning procedure), making a representational space for different face identities.

### Learning procedure

Learning occurs throughout areas in the visual system, especially in higher order areas^52^,^53^,^54^,^55^. Likewise, computational models adapt the wiring of layers to the statistics of input stimuli using learning mechanisms. In our proposed model of face processing, learning occurs in three layers: S2, VSL, and ISL. S2 layer simply learns a dictionary of prototypes (face parts); learning in the next two layers is based on a modified trace rule (in VSL) and a continuous invariant learning (in ISL).

Learning starts with tuning of S2 units (with prototypes of four sizes: 4, 8, 12, 16), using an unsupervised random selection mechanism from training images. In the next steps, the model uses a combination of two learning mechanisms: a modified trace rule^56^ and adaptive resonance theory (ART–38,57) to modify connection weights between C2 and VSL; as well as VSL and ISL.

### Learning a dictionary of face parts in S2 layer

During the learning phase, each unit in the S2 layer becomes selective to face parts, while training face images are being presented to the model. In every presentation of a face image, several S2 units become tuned to the image parts that fall within their receptive fields. These parts are mostly face components such as eye, nose, mouth, and/or combinations of them^40^. Responses of S2 units (1000 units) are maximal when the new input image matches the learned patterns. These units model the functional properties of neurons in the PL face patch in monkeys.

### Continuous view-tolerant learning rule in VSL & ISL

In everyday life, we continuously perceive various views of a person’s face. Therefore, adjacent face views are continually perceived across time. It seems that the visual system uses this characteristic as a clue to construct view-tolerant representation of faces (view-tolerant means that the representation remains relatively unchanged after changing a face view). Consistently, we proposed a learning mechanism to create a view-tolerant face identity representation in the model. We thus applied adjacent face views to the model continuously. Trace learning rule allowed us to create separate sub-population of units with tolerant properties. The response of an active unit consists of two parts: 1) the effect of previous inputs in the response (trace); 2) the effect of current input. The first part, which is constructed using trace rule, wires active units to each other and as a result, views of a person is coded through a linked sub-population of units.

The learning occurs simultaneously in the last two layers (i.e., VSL and ISL). The response function for the VSL units is shown in equation 2:


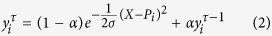


Where, *P*_*i*_ is the *i*^th^ template saved as the kernel of a Gaussian function, ρ is the vigilance parameter (threshold) that the model uses to decide whether adding a new unit to the layer or not, σ defines the sharpness of the tuning, which is set to a constant value (σ = 0.5) in a separate evaluation phase, and α is a trace coefficient that adds previous activity to the current output (α = 0.3). The term α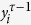 determines trace (memory) from previous responses.

To find out whether the learned unit is sufficient to represent the input, it is compared with ρ that determines the degree to which the unit properly represents the input; the optimal value for ρ is set in the evaluation phase –using a non-overlapping set of stimuli used only for evaluation. If the activity of the learned unit is lower than ρ (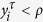), the learned unit has a poor representation of the input; so, a new unit is added to the VSL population that represents the input (see details in model evaluation part).

At the same time, a new connection between the VSL unit and the winner ISL unit is established using modified trace rule. These connections are developed through the learning process and build the tolerant face identification space. For example, different face views of an identity create almost the same pattern of activities in the ISL feature space. There is thus a particular representation for each identity that can be easily distinguished from others. The response function for the ISL units is calculated by equation 3:


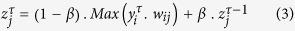


Where 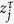 is the response of the *j*^th^ ISL unit at time τ, 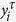 illustrates the activity of the previous layer. ISL function consists of two parts: 1) The initial part that applies a maximum operation to its inputs, with 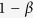 as the coefficient; 2) The trace part that includes previous synaptic activities, with 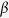 as the coefficient. The connection weights (*w*) between the ISL and VSL are binary. In the learning phase, when a new VSL unit shows a significant response greater than vigilance parameter (ρ), the unit is connected to the winner ISL unit. Thus, the weight between these two units is set to 1 (*w*_*ij*_ = 1). Equation 4 summarizes how the weights between VSL and ISL are learnt:


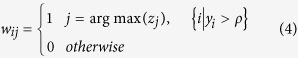


### Model evaluation

Images in the learning phase are sequentially presented to the model, 50 identities each in 37 views, starting with all views of an identity in random order and continuing to other identities. In order to avoid any learning bias to specific face views, while images are presented to the model, the first view of every identity is randomly selected and then other views (36 views) are presented in a sequential manner (e.g., if the first view is 45°, the next views are 50°, 55°, and so on).

The first image is applied to the model. Then, if there is not any unit in VSL, a unit with a Gaussian-like function, which is tuned to the input stimuli, is created. The second input image is subsequently presented to the model. Depending on the similarity of the input with the unit’s preferred stimuli, a new unit can be added to the VSL and correspondingly a connection is formed between this unit and another unit in ISL. After presenting all images of an identity (different views) a blank gray image is presented to the model. This blank gray image does not generate any activity in the units (baseline); therefore, all ISL units become silent until the next input is presented to the model. As a result, previous activities do not affect new input images and the trace, especially in the last two layers, is removed.

After each step in the learning phase (i.e., whenever a new unit is added to ISL layer), we have an evaluation phase to test the model discriminability between new identities. For this purpose, we use an evaluation dataset. The dataset contains 740 face images (20 identities, each in 37 views) that travel through the model’s hierarchical structure and produce different patterns of activities, especially in the last layer. Finally, the discriminability between identities is measured and compared to the previous state of the model (before adding new units), using a View-invariant Identity Selectivity Index (VISI), which measures identity selectivity, and a support vector machine (SVM) classifier to calculate identification accuracy in invariant face recognition. The VISI value is compared with a threshold; a value less than the threshold indicates that the new modification (units added to the model) had no significant impact on improving the discriminability. Therefore, the new added units are removed. As the representational space is developed, the learning process is saturated (i.e., goes from coarse to fine), and only a few units will be added to the model, Fig. 1. An SVM classifier is also trained on 18 face views of 20 identities of evaluation dataset (randomly selected from 37 face views) and tested on 19 face views. As shown in Fig. 1C, the identification accuracy is saturated during the learning procedure. When the learning procedure finishes, the parameters become fixed and does not alter in further experiments.

### View Selectivity Index

**Figure 2:**
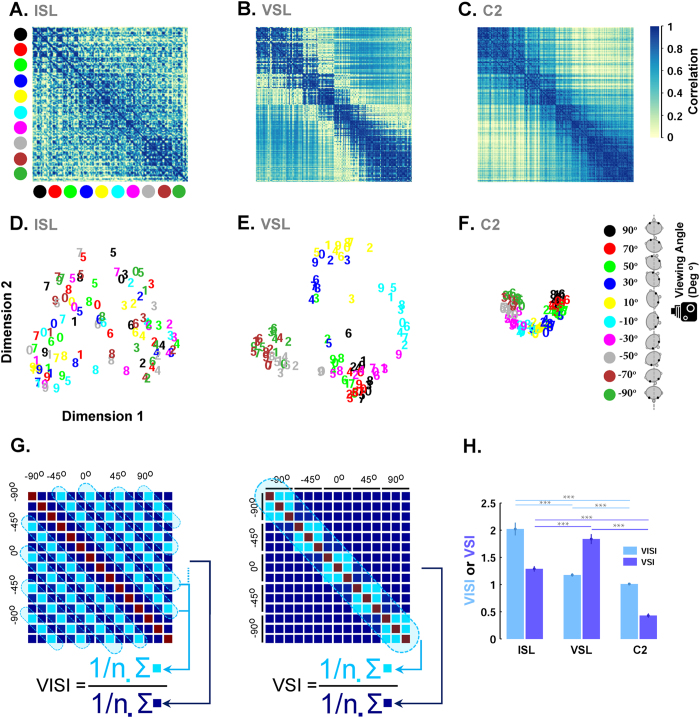
Representational geometries of face views and identities in ISL, VSL, and C2. Top row (**A–C**). Similarity matrices computed based on activities in ISL, VSL, and C2, from left to right, respectively. For each of these layers, a 100 × 100 similarity matrix was constructed by calculate the pairwise correlation (Pearson’s correlation) between the extracted feature vectors for 10 sample subjects in 10 sample face viewpoints (viewpoints are in the steps of 20° from −90° to 90°). Bottom row (**D–F**): Each panel depicts the results of multidimensional scaling (MDS) for responses to the face images in different layers (**D**: ISL, **E**: VSL, and **F**: C2). Each plot shows the location of 10 subjects (indicated by numbers from 1 to 10) at 10 face views (indicated by 10 different colors, shown in the right inset) for the first two dimensions of the MDS space. Note that the clusters of the face views and face identities are formed in VSL and ISL, respectively. (**G**) The method for calculating view selectivity index (VSI) and view-invariant identity selectivity index (VISI) are shown in this part. Pale blue values are divided to dark blue values. The diagonal line is omitted from the calculations. (**H**) VISI is significantly higher in ISL compared to VSL and C2 (ranksum test, p = 0.001). Face views are better decoded in VSL compared to ISL and C2 layers.

To calculate view selectivity index, a similarity matrix^58^ was computed from responses of three last layers. We then computed “view selectivity index” as follows: For each 740 × 740 similarity matrix (X) for test images (20 identities in 37 views), we computed the mean correlation along the squares (20 × 20) around the main diagonal of X and divided by the average of other parts of the matrix (Fig. 2G). The values of the main diagonal were omitted from the calculation because the correlation is always one on the diagonal.

### View-invariant Identity Selectivity Index

To calculate view-invariant identity selectivity index, a similarity matrix was computed from responses of ISL units. We then computed “view-invariant identity selectivity index” as follows: For each 740 × 740 similarity matrix (X), we computed the mean correlation along the off-center diagonals {y = x + 20, y = x + 40,… y = x + 720} of X and then divided by the average correlation values of other elements of the matrix (Fig. 2G). The values of the main diagonal were omitted from the calculation because the correlation is always one on the diagonal.

### Degree of Invariance

In the canonical face view experiment, we defined degree of invariance (DoI) as an index. This index indicates to what extent the ISL and C2 features are tolerant (invariant) to changes in views of an identity. View tolerance was measured by estimating a tuning curve obtained from comparing each viewpoint with other viewpoints using Pearson correlation. First, we calculated the pair-wise correlations between feature vectors corresponding to different identities at a selected viewpoint. Then, the average correlation value was considered as a threshold. Then, we calculated the correlation between the features vector of an identity at a given viewpoint and the features vectors obtained for different viewpoints of the same identity. Using a pixel-wise comparison the same viewpoint of two identities seems to be more correlated than two viewpoints of the same identity^59^. Therefore, if the correlation between different viewpoints of the same identity was significantly higher than the threshold correlation, the feature vector of selected viewpoint is tolerant to the other viewpoint. This procedure was done for every viewpoints of 20 identities in 10 independent runs. Finally, we report the number of viewpoints (degree) which are significantly higher than threshold.

### Image data sets

To evaluate the model in different experiments, we used several face image datasets. All datasets are widely-used face image datasets that are freely available. We provide a brief description about each dataset in the following sections.

### NCKU Face

We used NCKU dataset as a major face image dataset to train the proposed model since it contains face images with a precise variation in views. The database contains 3330 images of 90 subjects. There are 37 images, taken from 37 different viewing angles, for each identity. The viewing angles change from +90° (right profile) to −90° (left profile), with steps of 5°. The dataset is freely available on: http://robotics.csie.ncku.edu.tw/Databases/FaceDetect_PoseEstimate.htm^60^.

### Face Place

This face database was created by Tarr lab^61^. It has been used in experiments studying other race effect. We tested the model using the Asian and Caucasian races (similar to ORE psychophysics experiments:^23^,^24^,^62^. This part of the database includes images from 38 individuals of two races with consistent lighting, multiple views, and real emotions. Images of each identity come in seven views (+90°, +60°, +30°, 0°, −30°, −60°, −90°). The dataset is freely available through: http://wiki.cnbc.cmu.edu/Face_Place.

### Composite face stimuli

The Composite face stimuli^17^ have been built with the purpose of investigating the composite face effect in psychophysical and neurophysiological studies. There are images of 10 different identities and 5 compositions per condition (aligned and misaligned), resulting in 50 different images in each condition (100 images in total). In aligned face images, the upper half of a face image of an identity is combined with five different lower halves in a normal face configuration. In the misaligned condition, there are similar combinations with aligned faces, but upper and lower halves do not make a normal face configuration. The dataset is freely available on: http://face-categorization-lab.webnode.com/resources^17^.

## Results

Different layers of the model were analyzed; and model responses were compared with psychophysical data in humans and cell recording data in monkeys. The model performance and its similarity to biological data were assessed using representational similarity analysis (RSA-58).

### Representation of face views and identities in the network

Views and identities of different face images are represented over the last two layers of the network. Figure 2 shows response properties of the three last layers (C2, VSL, and ISL), visualized using multidimensional scaling (MDS), similarity matrix, and two indices of view and identity selectivity (VSI and VISI, see Materials and Methods). ISL responses show clear selectivity to identities when the model is presented with different views of an identity. Figure 2A visualizes this effect as parallel diagonal lines shown in the similarity matrix (similarity measured as Pearson’s correlation). The VSL similarity matrix (Fig. 2B) is characterized with a high similarity around the main diagonal, indicating view-specific representation, but no clear identity selectivity (parallel diagonal lines similar to ISL). Responses of VSL were highly selective for face images compared to other objects. Also, different populations of neurons represent different face views (Supplementary Fig. S4). A moderate degree of view-specific responses can also be seen in the activities of C2 layer, like VSL, with no selectivity for identities (Fig. 2C). MDS is a visualization method, which transforms data from a high dimensional space to a lower dimensional space^63^,^64^. The MDS plot (Fig. 2D) shows that each identity is clustered together in ISL (for 10 sample subjects, the numbers shows identities and different colors are used for different views). On the other hand, each cluster in VSL (Fig. 2E, different colors) represents a face view while identities are intermixed. In contrast, in the C2 space (Fig. 2F), views and identities are densely distributed and highly overlapped with each other, meaning that C2 responses are not sufficiently informative about views and identities. Similar results can be seen in the plots of VSI (View Selectivity Index) and VISI (View-invariant Identity Selectivity Index), as two quantitative indexes for the representations, Fig. 2H. Overall, C2 shows a slight selectivity for face features whereas VSL and ISL demonstrate view selectivity and identity selectivity, respectively. The response properties of three last hierarchically organized layers of the model highly resemble the responses of face patches in monkeys’ IT cortex –from posterior to middle and anterior face patches^4^.

### Invariance to face views

Behavioral studies have shown that canonical face view, a face view between frontal and profile views, have the highest information about the face identity^30^. We investigated whether a particular face view^30^,^31^,^32^ has a higher recognition performance compared to the other face views (such as full-face or profile). To this end, we used correlation analyses as well as identification performances.

**Figure 3:**
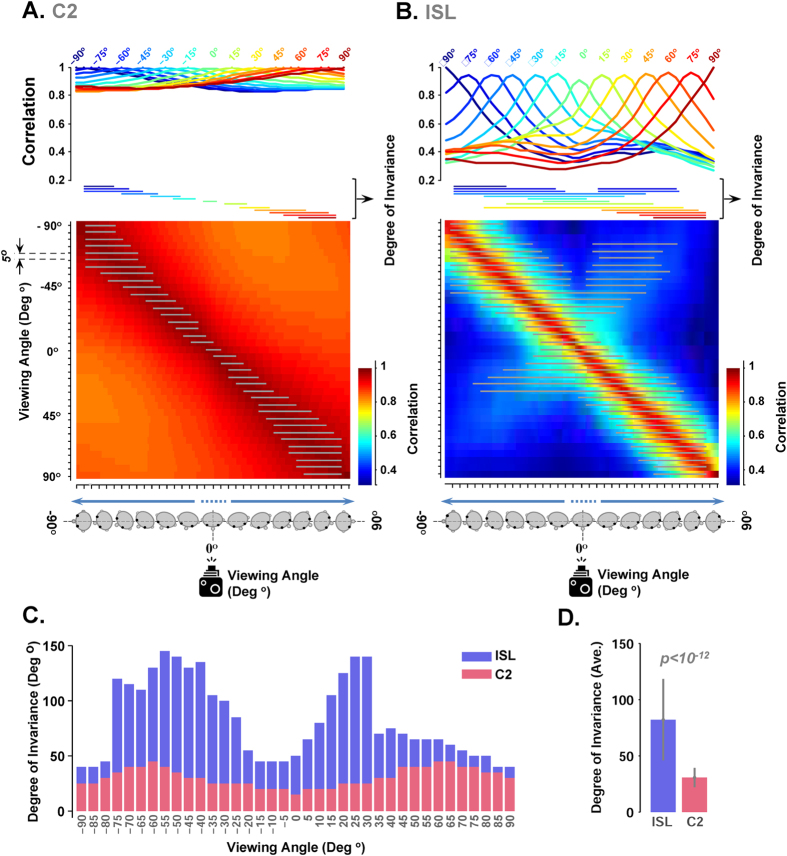
Higher degree of invariance (DoI) in ISL compared to C2. (**A**) View-tolerance at the level of C2 units. Each tuning curve shows the degree of invariance in the responses of C2 units for a particular viewing angle (face view). Only a subset of tuning curves is presented (details for every view is shown in Supplementary Fig. S1). The vertical axis is the correlation between feature vectors at one reference view from a set of subjects and feature vectors, computed for the same subjects across different view. The horizontal axis indicates different views with the steps of 5°. The colored, horizontal lines underneath each curve demonstrate the significant range of DoI (p < 0.02– ranksum test) for a particular view. Each row in the invariance matrix, below the tuning curves, corresponds to a tuning curve for a face viewpoint (viewing angles are separated by 5°, from −90° in the first row to +90° in the last row. Head poses and camera position are schematically shown along the horizontal axis). Color bar at right inset represents the range of correlation. The gray horizontal lines, printed on the invariance matrix, exhibit the degree of invariance for every view similar to tuning curves (ranksum test). (**B**) View tolerance at the level of ISLs. (**C**) Summary of view tolerance responses for each face view in C2 units and ISLs. Each bar exhibits the DoI for a face view for C2 units (red bars) and ISLs (blue bars). The horizontal axis shows different face views. (**D**) Average DoI across all views for ISL and C2, calculated using data shown in (**C**).

Figure 3 shows the comparison between responses of C2 and ISL units in terms of degree of invariance (DoI). C2 and ISL responses are quite different in their DoI value. To evaluate the tolerance properties of the ISL and C2 features, we used a methodology similar to^65^; see also:^66^,^67^,^68^,^69^. View-tolerance was measured by first estimating a “tuning curve”, obtained by correlating a feature vector corresponding to one face image at a given view with a feature vector for the same subject at different views (37 face views with the steps of 5° from −90° to 90°). An average tuning curve was then obtained by averaging similarities across views of subjects and over 10 random runs, 20 sample identities for each run. The level of invariance for each face view was determined by computing its correlation with other views of the same identity, and then averaging across the correlations; if the average was significantly higher than a pre-defined threshold, then that view has an invariant representation. The threshold is calculated for each face view by computing the maximum correlation between the feature vectors of all subjects at the same view. A tuning curve was calculated for each face view based on the activities of C2 and ISL (37 views, 37 curves–see Fig. S1), representing the degree of invariance for these layers. Several samples of tuning curves are shown in Fig. 3. The invariance matrices (Fig. 3A,B) show the regions in which the correlation between views is significantly higher than the invariance threshold, meaning that those views carry a higher amount of view-tolerant information of an identity. Consistent with behavioral studies^30^,^31^, we see a high degree of invariance in canonical views (Fig. 3C). Interestingly, this effect is more dominant in ISL compared to C2 (Fig. 3C). The DoI of ISL features is significantly higher than C2 features across all face views (Fig. 3D– p < ten power minus twelve (10e-12), ranksum test).

**Figure 4:**
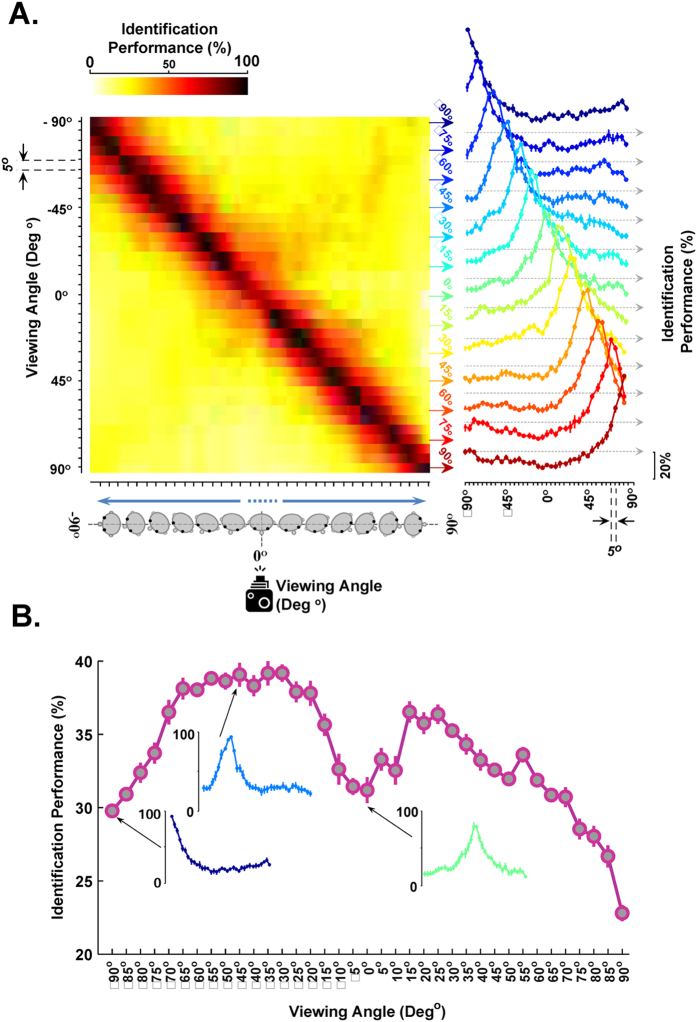
Performance of the model (ISL) in view invariant face recognition. (**A**) The performance of face identification in different views. The color-coded matrix shows the performance of the model in identifying subjects across different views. Each row of the performance matrix illustrates the performance of the model for one view (trained using a particular view and tested over all views). The color bar at the top-left shows the range of identification performance. The vertical axis shows different face views for training. The horizontal axis corresponds to different test views, the first row of the matrix shows that a classifier trained by −90° and tested with all other views. The chance level is 5%. A subset of performance curves is shown at the right inset, demonstrating the performance variations in different views, the peaks of performance curves change as the training views change (details of performances in every view are shown in Supplementary Fig. S2). The small, black, vertical axes at the right of the curves show 20% performance. Error bars are standard deviations over 10 runs. (**B**) Performance comparison across different views. Each circle refers to the average of recognition rate in each view (i.e. the mean performance across all views). The vertical axis indicates the mean performance and the horizontal axis shows different views. Several performance curves are shown for some sample views. Error bars are the standard deviation and the performances are the average of 10 runs.

We also analyzed the performance of the model in invariant face identification using ISL features using support vector machine (SVM) classifiers, Fig. 4. The SVM was trained with one view and tested by other views (repeated across 10 individual runs for every view, separately). The performance decreases as the views deviate from the training view, Fig. 4A (see Supplementary Fig. S2 for more details). This observation might not be surprising; but, the interesting point is that the degree of invariance in ISL features increases around canonical face views, Fig. 4B. These evaluations exhibit that the model is able to represent the effect of canonical face views (views that contain more tolerant information)^30^,^31^,^32^.

### Only ISL feature space has a dominant face inversion effect

**Figure 5:**
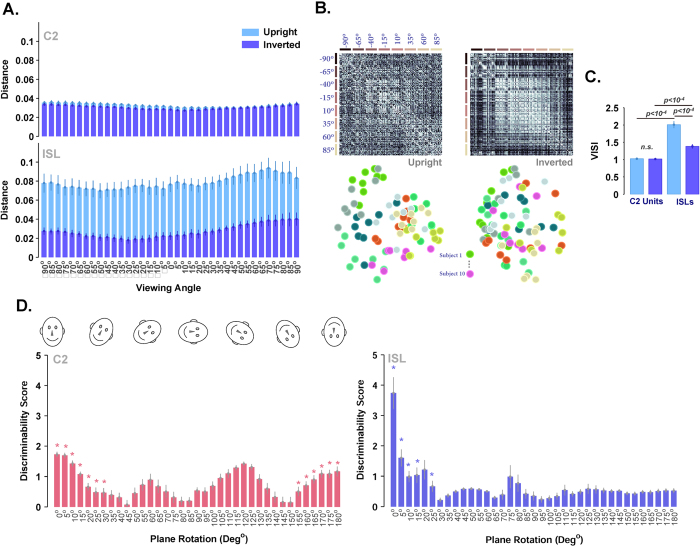
Face inversion effect (FIE) for different views. (**A**) The distance between feature vectors of inverted and upright face images for C2 units (up) and ISL (down). Inversion effect is highly significant at ISL compared to the C2 layer (normalized Euclidean distance). The vertical axis indicates the normalized distance and the horizontal axis shows different views, separated with the steps of 5°. The cyan bars represent the results for upright face images and the purple bars show the results for inverted face images. (**B**) MDS similarity matrices in the ISL upright (left) and inverted (right) faces. Similarity matrices show the pairwise similarities between the internal representations of the model for two different face views. The diagonal, parallel lines in the similarity matrix for upright faces (left) indicate the identity selectivity in the ISL for upright faces. The similarity matrix for inverted face images is shown at right. The lines along horizontal and vertical axis indicate different face views. Left MDS shows the results for upright faces while the right MDS represents the results for inverted faces. Color-coded circles in the MDS space represent subjects (10 subjects) at eight different views. (**C**) VISI for upright and inverted faces in the model (ranksum test –see Materials and Methods). Error bars are the standard deviation (STD) obtained over 10 independent runs. (**D**) Discriminability score (i.e. z-scored mean pairwise Euclidean distance between identities in the same in-plane rotation) is computed between feature vectors of images for C2 units (left) and ISL (right). The vertical axis indicates the discriminability score and the horizontal axis shows different plane-rotations, separated with the steps of 5°. Sample plane rotations of a schematic face are shown at the top of C2 units’ responses. Stars show significant discriminability scores (one-sided ranksum test, FDR corrected at 0.05).

Face inversion effect (FIE) has thoroughly been studied both physiologically and psychophysically^5^,^19^,^70^,^71^,^72^,^73^. Subjects’ performances in face identification drop significantly when inverted faces are presented^28^. This effect is one of the widely-used stimulus manipulations to investigate face recognition mechanisms in the brain. Here, we evaluated responses of the model in face identification tasks when face images were either inverted or normal. We examined the inversion effect in two layers of the model: C2 and ISL. Figure 5 shows a clear inversion effect in ISL units; however, C2 features either do not show the face inversion effect or only show a very weak effect in a few face views.

We used four different approaches to investigate the inversion effect across layers of the model: measuring Euclidean distance between upright and inverted faces, computing similarity matrices across all views for both upright and inverted faces, MDS plots, and VISI. Average Euclidean distance between ISL feature vectors (averaged across the same view of all identities) is significantly higher for upright face images compared to inverted faces. Once inverted faces were fed to the model, the discriminability of the units dropped and identities seemed to be similar; therefore, the distance between representations is reduced. However, C2 features had no significant difference in their Euclidean distance for the two cases (upright/inverted), Fig. 5A. The diagonal lines in the similarity matrices and the pattern of distributions in the MDS plots were two measurements that enabled us to better investigate the inversion effect in the upright and inverted faces, Fig. 5B,C. Parallel diagonal lines in the similarity matrices of upright faces indicate that identities (10 sample subjects) are represented better compared to the inverted case in ISL (a subset of 8 face views are shown). Colors in the MDS plot, which represent identities, are clustered more strongly in upright faces than inverted (10 sample subjects, Fig. 5B).

Furthermore, using VISI (see Materials and Methods), we quantitatively showed the representations of identities in the model for upright and inverted face images, Fig. 5C. VISI is significantly higher in ISL units for upright compared to invert faces, meaning that ISL activities resemble psychophysical data in humans. In addition, VISI is significantly greater in ISL compared to the C2 units (Fig. 5C *– p *< 10^−4^, ranksum test).

In another FIE experiment we studied the effect of in-plane rotation of frontal face views, Fig. 5D. For this analysis, similar to the previous section, the model was trained using NCKU dataset (see Materials and Methods for details). For the test phase, frontal view images (0° depth rotation) of 20 identities were selected and a circle aperture is used around each face image. Face images were then rotated in plane from 0° to 180° with steps of 5° and fed to the model. C2 features and ISL features were computed for each face image. In each in-plane rotation, we measured pairwise Euclidean distances between 20 feature vectors extracted from the 20 identities. The Euclidean distances were z-scored and then averaged across all identities (we called this ‘discriminability score’). The procedure was done for 10 independent runs of the model. Using ISL features face identities can be discriminated (significant discriminability score) only after small in-plane rotation of faces (~up to 25°, Fig. 5D-ISL). With more sever in-plane rotation of faces, the discriminability between different identities was reduced and so the identities were perceived to be similar by the ISL features. This seems consistent with the results of the behavioural study by^74^ where subjects’ reaction time increases with increasing in-plane rotation from 0°–180°. In contrast, C2 features had significant discriminability scores at very small and large rotations, so the discriminability score for C2 features did not decrease monotonically (Fig. 5D-C2). This is suggestive of part-based processing in C2, as opposed to holistic processing. Interestingly in a behavioral study^75^ a similar pattern is reported for objects.

### Composite face effect in ISL

**Figure 6:**
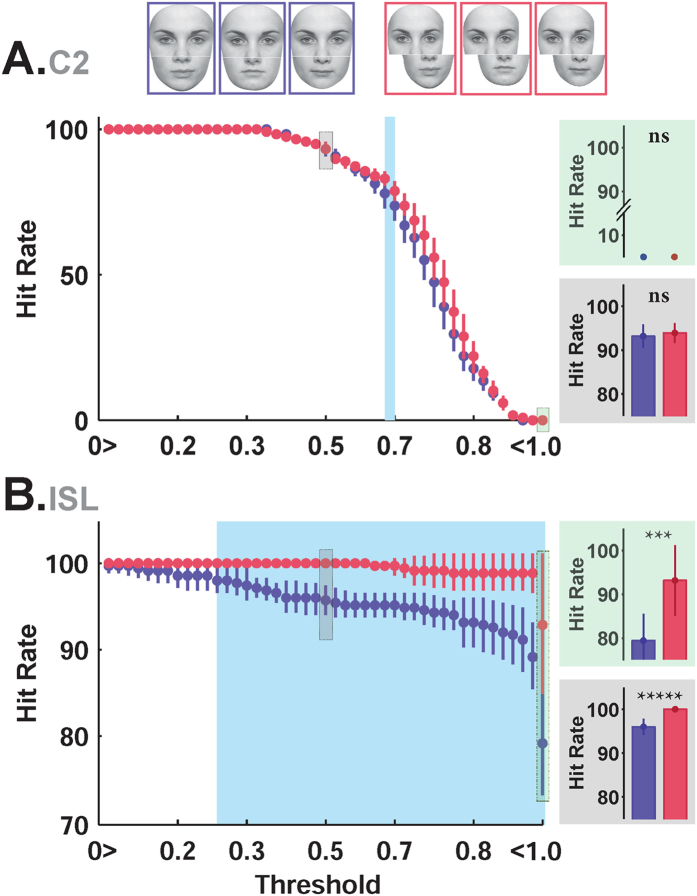
Model responses in the aligned vs. misaligned face identification task (Composite Face Effect). (**A**) The hit rate in identification of aligned (purple) and misaligned (red) faces in the C2 layer. The vertical axis shows the hit rate while the horizontal axis shows the threshold range (see Material and Method). Several samples of aligned (purple frames) and misaligned (red frames) face images^98^,^99^ are shown at the top of the plot. Two sample bar plots are shown at the right inset for two different thresholds: 0.5 (gray background) and ~1 (green background). The blue region is the area in which the hit rate between aligned and misaligned faces is significantly different (ranksum test). (**B**) The hit rate in identification of aligned and misaligned faces in ISL. In both A and B each point corresponds to the hit rate for the threshold value shown on the X-axis (different thresholds specify the boundary of the model to consider two face images as the same identity, 0 < thr < 1).

One of the interesting phenomena in face perception is Composite Face Effect (CFE). The effect is seen when two identical top halves of a face image are aligned with different bottom halves and their top halves are judged to be same or different (ignoring the bottom halves)^16^. Top halves are perceived as different identities, suggesting that face identification is done holistically and so the whole face image is needed for identification^17^. The visual illusion (mis-identification) disappears in misaligned composites, that is when a top half is slightly shifted to the right or left of the bottom half – not perfectly aligned on top of the bottom half^16^. Many studies have used this paradigm to illustrate that face perception is performed through the integration of different face parts as a whole, suggesting that the visual system processes faces holistically^16^,^17^,^18^. To assess whether the proposed model shows a composite face effect, we designed an experiment using composite face stimuli, and trained the model with faces from NCKU dataset^60^ (see Material and Methods for details). In the test phase, the model was presented with composite face stimuli from^17^, consisting of aligned/misaligned face images of 10 identities, each having five compositions. In each trial, two composite faces with identical top-halves were presented to the model and then C2 features and ISL features were achieved. Higher hit-rate (identification performance) for misaligned compared to aligned composites is defined as the composite face effect. For each identity, we measured the similarity between composite faces with the same top halves by first calculating their Euclidean distance, and then feeding it to a Gaussian function (*e*^*−d*^), where *d* is the distance. So the larger the distance, the lower the similarity. The hit-rate is then defined as the number of times that two composite faces with the same top halves are deemed similar divided by the total number of similar composite faces. Faces are deemed similar if their measured similarity (*e*^*−d*^) is larger than a pre-defined critical value (a threshold). We vary this similarity threshold from 0 to 1 (because *e*^*−d*^ can vary between 0 and 1); in each given threshold, the hit rate is computed in response to the aligned and misaligned faces (Fig. 6). As opposed to the C2 layer, ISL responses are not significantly affected by changing the threshold value (Fig. 6B); but in C2 the hit-rate significantly drops by increasing the threshold. For ISL, the hit-rate in misaligned images (red curve) is significantly higher than the aligned faces (purple curve) for all thresholds above 0.25. This indicates that two identical top halves with misalignment are assumed more similar than the aligned case (i.e. having two identical top halves aligned with different bottoms halves, which makes them to be perceived as different identities). The holistic representation of ISL units is able to account for this phenomenon. On the other hand, part-based units in C2 layer fail to show the composite face effect. There is no clear difference in C2 responses between aligned and misaligned faces, Fig. 6A. This suggests that face features in C2 are insensitive to the alignment/misalignment of composites. The patches in the C2 layer are independent of each other, in the sense that they are applied to different parts of the input image. Therefore, in the C2 layer faces are seen as a combination of different parts, but not as a whole. On the other hand, ISL features are computed by taking input from all these parts at a time and thus processing the input face as a whole. These differences at the level of computation makes these two layers behave differently.

### We better identify faces of our own race: Other-Race Effect (ORE)

People are better at identifying faces of their own race —within which they are grown up— than other races, an effect known as the other race effect (ORE–e.g.^23^,^24^,^62^,^76^). Similarly, we show in this section that the model better identifies faces of the race it is trained with.

Some studies suggest that there are different mechanisms for the identification of faces of the same and other races (e.g. holistic- versus component-based face processing–77. Here, we used two face image datasets of Asian and Caucasian races to assess this effect in the proposed model and compared the responses of the model with reported data from human psychophysics^17^.

**Figure 7:**
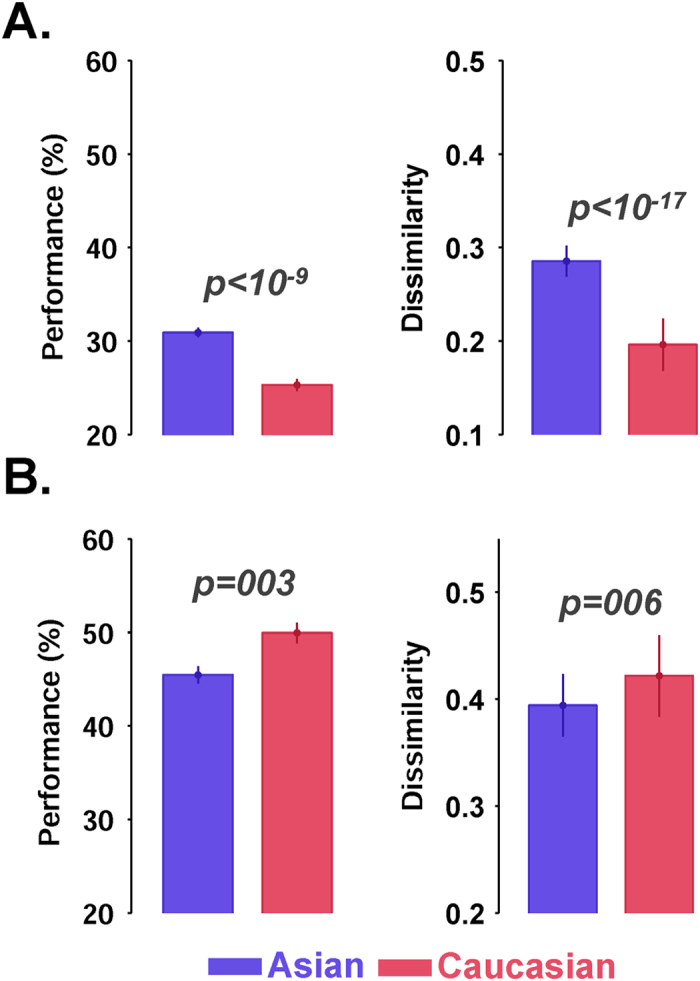
Discriminability of ISL units in response to Asian and Caucasian faces. (**A**) The dissimilarity (right- calculated based on Euclidean distance) and performance (left) between feature vectors of different races (using ISL features). A typical other-race effect can be seen, as observed in face recognition tasks in behavioral studies. ORE is highly significant in ISL. The model was trained using images from NCKU dataset (Asian race) and tested using Asian and Caucasian images from Tarr dataset. The vertical axes indicate identification performance (left) and dissimilarity calculated based on normalized Euclidean distance (right). The blue bar indicates the results for Asian face images and the red bar shows the results for Caucasian face images. (**B**) The dissimilarity (right) and performance (left) between feature vectors of different races in ISL when the model was trained on Caucasian faces and tested using both Asian and Caucasian (Tarr dataset). In all plots error bars are the standard deviation obtained over 10 runs. P-values calculated using ranksum test.

To test the effect, the model was first trained using Asian faces (from NCKU dataset) and tested on both Asian and Caucasian, Fig. 7A. Second, we investigated this effect by changing the races in the train (Caucasian) and test (Asian) phases, Fig. 7B. We trained the model with Tarr dataset on Caucasian^61^. The dataset contains 75 Caucasian identities. We train and evaluate the model with 55 Caucasian identities and test it with 20 Asian and Caucasian identities. ORE is shown using two measures: identification performance and dissimilarity. Identification performance was measured using a SVM classifier, trained on adjacent views (−90°, −30°, 30°, 90°) and tested on middle views (−60, 0, 60), for Asian and Caucasian face images, separately. Dissimilarity was measured by computing the average Euclidean distance within the faces of the same race. In both performance and dissimilarity, the discrimination between identities is significantly higher for the same race (ranksum test– *p *< 0.003; Fig. 7), confirming the reported behavioral results^23^,^77^. We further investigated the effect for each of the face views separately. In ISL, almost in all views (−90°, −60°, −30°, 0°, 30°, 60°, 90°) the dissimilarity is significantly higher for the same race compared to the other race (Supplementary Fig. S3).

## Discussion

We introduced a new biologically-plausible model for face recognition, consistent with recent cell recording data^3^,^4^. In particular, the model was able to account for response properties of face patches in monkeys as well as several well-studied behavioral phenomena for face processing in humans such as: face inversion effect, composite face effect, canonical face view, and other race effect. We considered both modern theories of face and object processing (i.e., population/distributed coding) and some classical, yet powerful, ideas (e.g., holistic face processing) in the model.

### Simulating the idea of specialized face processing

A fundamental question in biological object-vision is whether the brain utilizes the same mechanism to process all object categories or employs a specialized mechanism for particular categories (generic vs. specialized). The former is the generic view, suggesting that any object category is represented over distributed patterns of neuronal activities in the IT cortex. Objects can be discriminated based on distinctive patterns of activities^78^,^79^,^80^,^81^, the latter suggests that there are specific areas in the IT cortex highly selective to some categories, such as faces^3^,^4^,^5^,^8^, scenes^82^,^83^,^84^,^85^ and bodies^86^,^87^. Functional MRI studies show that evoked responses in other areas, excluding face selective regions, contain sufficient information for face/non-face discrimination^2^,^78^,^81^. Therefore, face selective patches are suggested to be involved in more specific tasks of face recognition (i.e., view-tolerant face identification^4^). Specialty of faces has also been demonstrated by several behavioral face specific phenomena^18^,^28^,^30^,^76^. Units of the proposed model are highly selective for face images but not for other objects (Supplementary Fig. S4). The proposed model is specialized for face processing, by design, and as such is comparable to some other biologically-inspired models (e.g. VisNet or HMAX) when only trained to recognize faces^38^,^88^. For example, Robinson and Rolls (2015) have compared HMAX and VisNet in processing scrambled vs. unscrambled faces. They show that VisNet neurons, as opposed to HMAX neurons, did not respond to scrambled faces (only responded to unscrambled faces). This supports the idea of holistic face processing, and is therefore consistent with our results here, where we also find, via composite face effect and inversion effect that features in the last layer of our model (ISL) process faces holistically. However, this behavior is not seen in a previous layer of our model, which corresponds to C2 features of the HMAX model. Holistic face processing is further discussed in the following subsections.

### Grandmother cells vs. distributed coding

The idea of grandmother cells emerged in the last two decades, indicating that there are highly selective neurons for particular objects/faces^89^,^90^. In this coding scheme, no further processing was required to extract an object label from neuronal representations. However, it seems implausible to have a separate cell for each object because it restricts the number of objects under consideration^91^. Distributed coding is the other side of the debate, suggesting that the information (e.g., face identity) is distributed over a population of neurons in higher visual areas. In this scheme each neuron is involved in representation of different stimuli. Therefore, none of them needs to be precisely tuned to a particular stimulus and an extra processing stage is required to readout the representations^91^.

Our model is designed in line with the idea of distributed coding; face views and identities are stored over a population of several units. For any given face image there are few responsive units in VSL; this is consistent with electrophysiological studies showing that face views are encoded sparsely^4^,^92^. Units in ISL represent face identities over a distributed pattern of activities, meaning that each unit is involved in encoding many identities and the response of a single unit is not solely informative enough about an identity. Consequently, information of an identity is distributed over responses of all units. The pattern of responses for an identity is also tolerant to different views of the identity.

There is infinite number of identities that need to be represented over the population of neurons in face selective areas. This requires a distributed sparse coding approach that enables encoding many identities by eliciting different patterns of activities in face selective areas. It seems that identities are less likely to be encoded using grandmother cells due to limited number of neurons that exist in face selective areas.

### Holistic face processing is only seen in the last layer of the model corresponding to AM

Several studies have suggested that faces are processed as wholes rather than individual parts, which is referred to as holistic face processing^77^,^93^,^94^,^95^,^96^,^97^,^98^,^99^. Disturbing the configuration of face images leads to reduction in both recognition speed and accuracy^94^,^97^. Many behavioral studies have evidenced holistic processing using various experimental procedures^17^,^23^,^73^. We tested our model in three different well-known face experiments, supporting the idea of holistic face processing. First, we investigated the *Composite Face Effect*. When two identical top halves of a face image are aligned with different bottom halves, they are perceived as different identities and we are unable to perceive the two halves of the face separately. ISL units in our model –which correspond with AM face patch— showed a similar behavior: the dissimilarity was higher between aligned face images than misaligned faces. This is because this layer of the model, which is the last layer, represents face images holistically and has misperception when encounters with aligned images. Second, the model shows a face *Inversion Effect*, another well-studied effect, supporting holistic face processing. Performance drops when inverted faces are presented to humans^100^,^101^,^102^. Upright face images are processed using configural and featural information (holistic–97, which is also regarded as evidence for multi-feature selectivity^103^). The face discriminability of the model was reduced when inverted face images were presented. Finally, the model also showed another face-related phenomenon, known as the *Other Race Effect*; again, a perceptual effect confirming holistic face processing. It is suggested that the holistic processing of face information occur for face images of our own race, which enables us to better identify individuals who have a face more similar to the average face we have as a template^23^.

We showed that ISL units in the model have properties such as, composite face effect and inversion face effect, suggesting that faces are processed holistically in this layer. However, this is not an obvious feature of the C2 layer, which is considered as a part-based layer in the model analogous to PL in monkey face patches (having similar representational geometries for both upright and inverted face images; Fig. 5 suggests that the C2 layer is not sensitive to holistic information such as configuration). It suggests that the C2 layer is rich enough for object recognition and face/non-face categorization, but not for face view and identity coding.

Our results in the IE experiment shows that the inversion effect happened for all views (see Fig. 5). This suggests that all views are processed holistically; behavioral studies have also shown similar results in humans^104^,^105^.

### Where does the holistic perception of face stimuli emerge in the brain?

We investigated several behavioral face effects in the proposed model that have been reported in the literature. The question is whether these effects originate from stimulus statistics or the visual system? Many studies in the face literature suggest that the effects are specific for faces compare to other objects. Therefore, the effects can be divided into two groups: those that are intrinsic to the stimulus and those that are cognitive and are due to the way that visual information are processed in the brain. Several models may satisfy the stimulus-dependent effects that are mostly originated from statistics of faces. For example, in our model even the early layers (up to C2) can capture these stimulus-driven effects. However, not every model is able to satisfy the cognitive effects, which are dependent on the specific learning mechanism in the model that has mimicked the face-processing network in the brain. For instance, canonical effect was shown in the C2 layer (which is effectively output of the HMAX model) and also ISL layer. But, the composite face effect, which is considered to be a cognitive effect, only emerged in the ISL layer. Meaning that a simpler model of visual information processing (i.e. HMAX model that corresponds to the C2-layer of our model) does not explain the composite face effect phenomena. These results suggest that higher-level cognitive effects, such as composite face effect, and more generally the holistic perception of faces emerge in higher layers of primate’s face processing system (e.g. anterior face patches).

## Additional Information

**How to cite this article**: Farzmahdi, A. *et al*. A specialized face-processing model inspired by the organization of monkey face patches explains several face-specific phenomena observed in humans. *Sci. Rep.* **6**, 25025; doi: 10.1038/srep25025 (2016).

## Supplementary Material

Supplementary Information
