# Supplementary material for: A specialized face-processing model inspired by the organization of monkey face patches explains several face-specific phenomena observed in humans
Source: Sci Rep. 2016 Apr 26;6:25025. doi: 10.1038/srep25025 (PMC4844965; doi:10.1038/srep25025)
Supplement: Supplementary Information [file srep25025-s1.pdf]

A specialized face-processing model inspired by the organization of monkey face patches explains several face-specific phenomena observed in humans

Amirhossein Farzmahdi[1], Karim Rajaei[1], Masoud Ghodrati[2], Reza Ebrahimpour[3,1*], Seyed-Mahdi Khaligh-Razavi[4*]

1 School of Cognitive Sciences (SCS), Institute for Research on Fundamental Sciences (IPM), Tehran, Iran
2 Department of Physiology, Monash University, Melbourne, VIC, Australia
3 Department of Computer Engineering, Shahid Rajaee Teacher Training University, Tehran, Iran
4 Computer Science and Artificial Intelligence Laboratory, Massachusetts Institute of Technology, USA

* E-mail: Corresponding ebrahimpour@ipm.ir, skhaligh@mit.edu

SUPPLEMENTARY INFORMATION

Supporting Text 1. Face Selectivity in VSL: To evaluate the response properties of VSL units, we assessed selectivity of the units for face and object images. We selected eight diverse categories of objects from Caltech-256 dataset [1] (consisting of the human face, animal body, fruit, gadget, human body, animal face, plant, and scramble images; 16 images for each category) and applied them to the model. Each row of the scatter plot in Figure S4 (top) shows the mean response of a VSL unit across all (8×16) images. Color-coded values show the amount of normalized activity. As shown in Figure S4 (top), high responses of units for face-like images declare face selective responses. Furthermore, the VSL units have selectivity to specific face views, Figure S4 (bottom). These two properties resemble response characteristics of the population of ML/MF neurons (for comparison with electrophysiological data see Figure 3 in [2]).

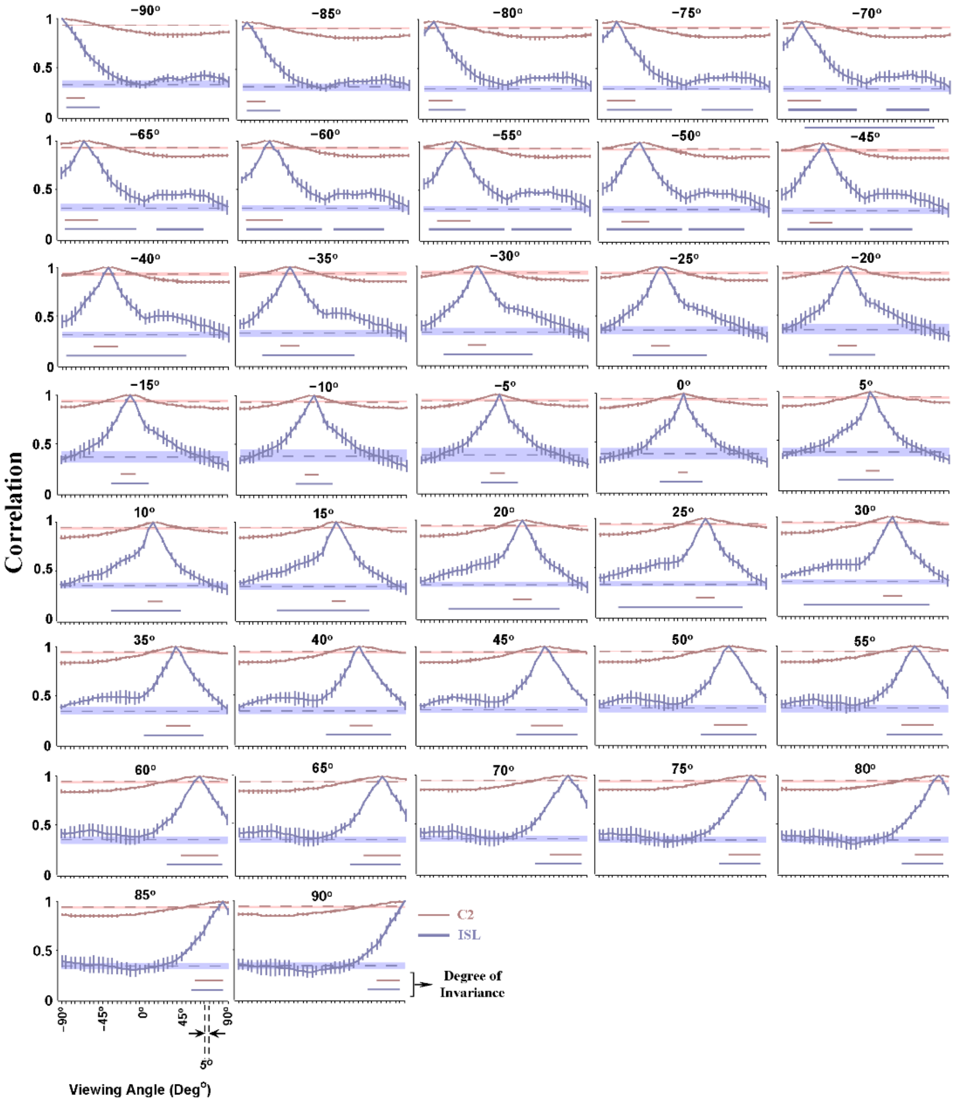

Fig. S1. View invariant tuning curves for C2 and ISL units across different face views. The pink curves indicate tunings for C2 units and the purple curves indicate tunings for ISL units. Each point on a curve exhibits the correlation between feature vectors at one reference view from a set of subjects and feature vectors computed for the same subjects across other views. The horizontal axis shows views, separated with the steps of 5º. The horizontal dashed lines, shaded with error area, show the average correlation among feature vectors in one view of different subjects. Error bars are the standard deviation and the correlations are the average of 10 random runs. The horizontal lines, underneath the curves represent the degree of invariance for C2 responses (pink lines) and ISLs (purple lines).

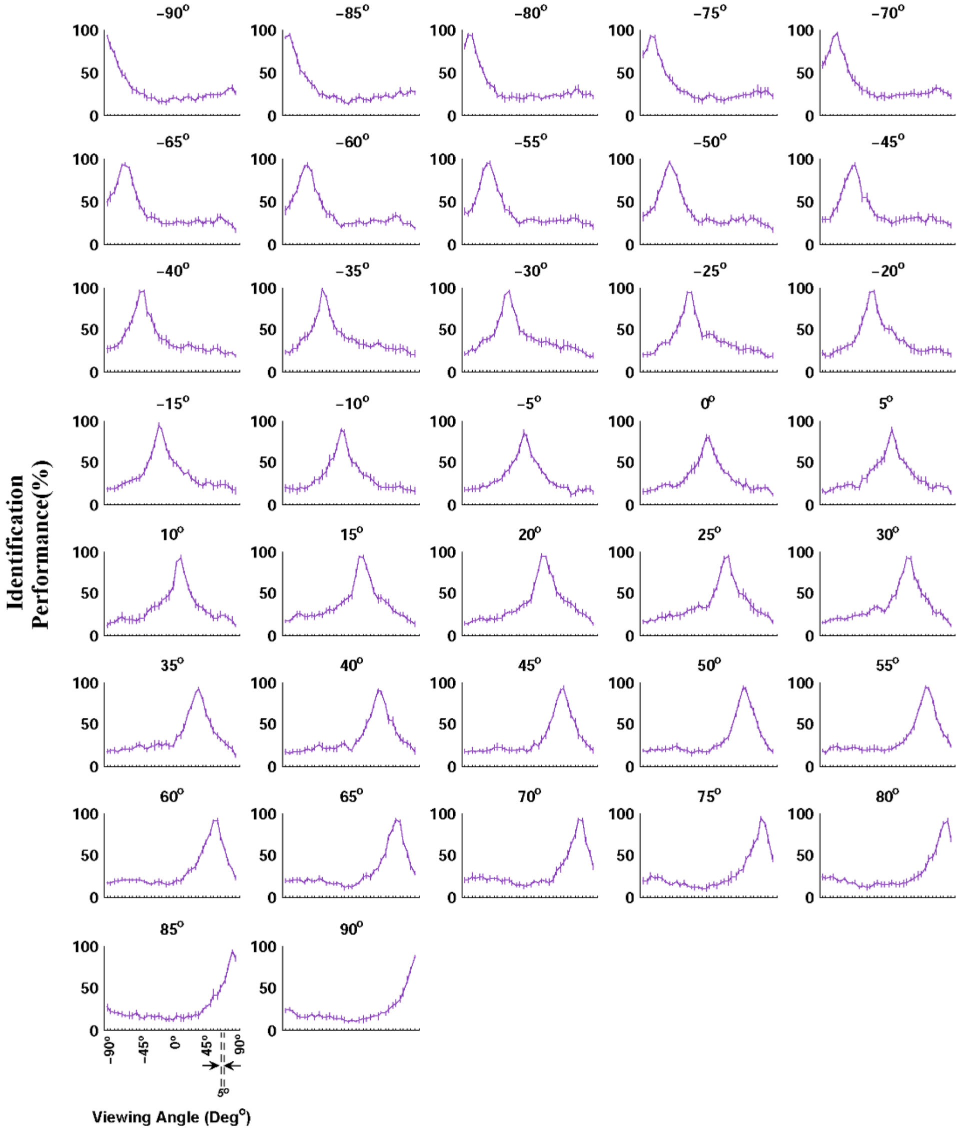

Fig. S2. Performance of the model in face identification for the ISL. Each plot shows the performance of the model for a face view. Each point on a curve (a sample train view) shows the identification performance at one test face view from a set of subjects, 20 identities. The vertical axis indicates the identification performance and the horizontal axis shows different views separated with 5º. Error bars are standard deviations, and performances are the average of 10 runs.

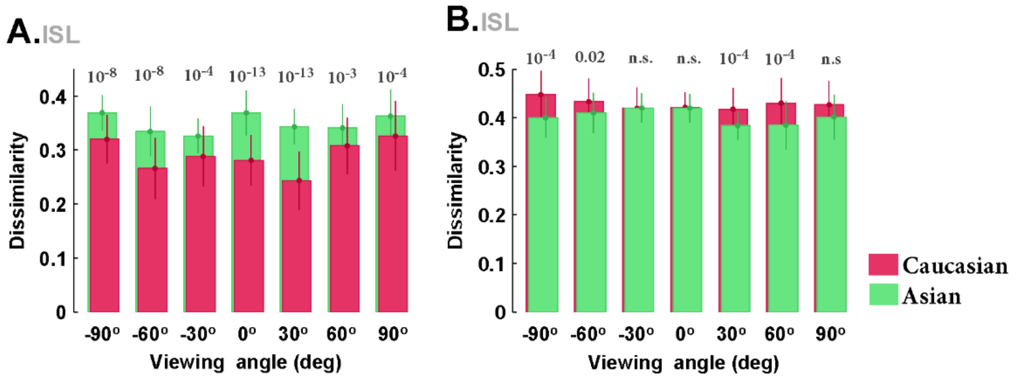

Fig. S3. Illustration of the other-race effect in ISL across different views. A. The dissimilarity for own- vs. other-race faces as a function of face views when the model is trained by Asian and tested on both Asian and Caucasian . The horizontal axis shows seven views (+/- 90 º, +/- 60 º, +/- 30 º, 0 º) and the vertical axis indicates dissimilarity. Simulations show a decrease in dissimilarity (as a result of ORE)**. B.** The dissimilarity for own- vs. other-race faces as a function of face views when model trained on Caucasian and tested by both Asian and Caucasian in ISL. In all plots error bars indicate standard error of the mean, and dissimilarities are the average of 50 independent runs within each race. *P-values* were calculated using ranksum test.

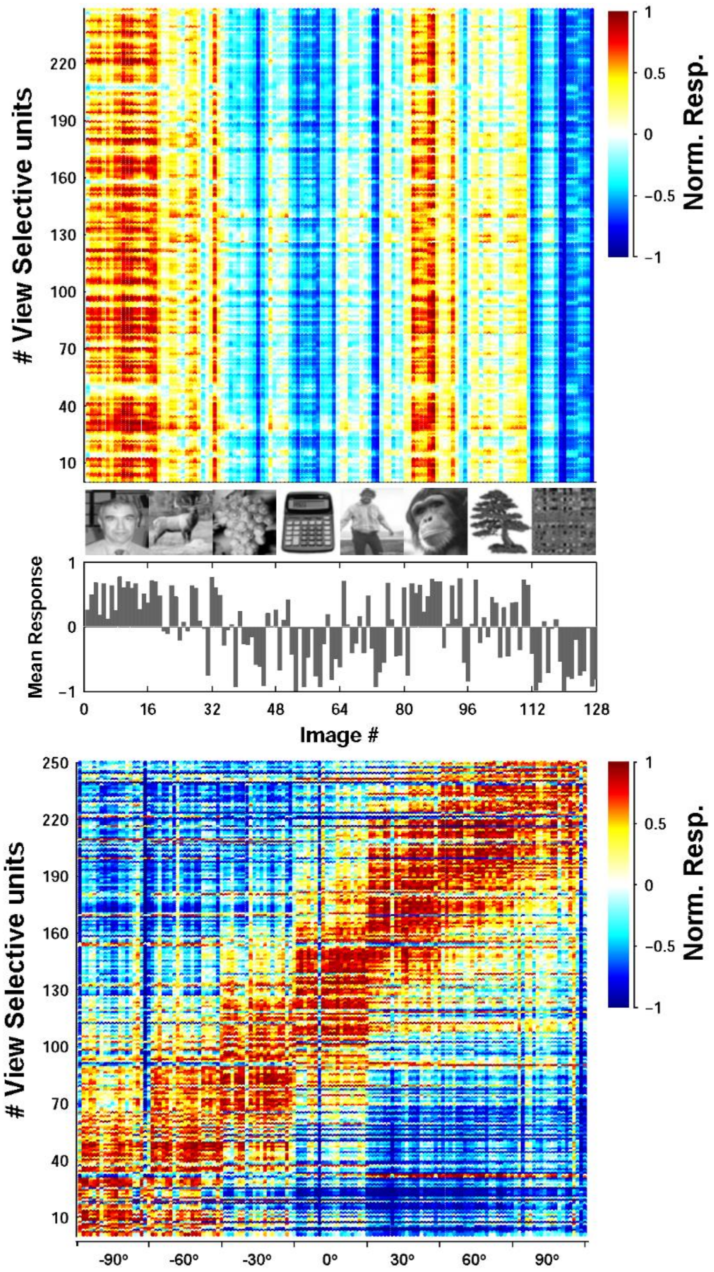

Fig. S4. Face selectivity in VSL units. Top. VSL responses to eight categories of objects. The mean response of the units is shown in the bar plot, below the figure, the normalized activity is color-coded. The horizontal axis shows eight object classes with 16 images per class from Caltech-256 dataset [1] (Human face, Animal body, Fruit, Gadget, Human body, Animal Head, Plant, Scramble images) and the vertical axis indicates the normalized response of VSL units. **Bottom**. Responses of VSL units to different face views. The horizontal axis shows seven sample views for an identity. Vertical axis depicts responses of VSL units. Stimulus images courtesy of Michael J. Tarr, Center for the Neural Basis of Cognition and Department of Psychology, Carnegie Mellon

University, http://www.tarrlab.org/. Funding provided by NSF award 0339122. (http://wiki.cnbc.cmu.edu/Face_Place).

References

1. Griffin, G., Holub, A. & Perona, P. Caltech-256 object category dataset. In *CNS-TR-2007-001* (2007).
2. Freiwald, W. A. & Tsao, D. Y. Functional compartmentalization and viewpoint generalization within the macaque face-processing system. *Science* **330,** 845–851 (2010).
